# Digital music and movement intervention to improve health and wellbeing in older adults in care homes: a pilot mixed methods study

**DOI:** 10.1186/s12877-024-05324-3

**Published:** 2024-09-04

**Authors:** Len De Nys, Esther F. Oyebola, Jenni Connelly, Gemma C. Ryde, Anna C. Whittaker

**Affiliations:** 1 Faculty of Health Sciences and Sport, University of Stirling, Stirling, Scotland UK; 2 School of Cardiovascular and Metabolic Health, University of Glasgow, Glasgow, Scotland, UK

**Keywords:** Older adults, Physical activity, Digital health intervention, Anxiety, Fear of falling, Loneliness, Physical function, Cortisol, DHEA, Pilot study

## Abstract

**Background:**

Low physical activity among older adults is related to adverse health outcomes such as depression and loneliness, poor physical function and increased risk of falls. This study was designed to increase physical activity through a digital, group-based, physical activity and music intervention and to examine its effectiveness on social, mental and physical health outcomes.

**Methods:**

Participants were 34 older adults (65 years +) recruited across four care homes in Scotland to a pilot study. Surveys were administered at baseline and post-intervention, comprising measures of fear of falling, depression and anxiety, loneliness, sleep satisfaction and quality of life. A battery of physical function tests and saliva sampling for cortisol and dehydroepiandrosterone hormone analysis were also conducted at each time point. Additionally, process evaluation measures (recruitment, intervention fidelity, attendance, retention rates and safety) were monitored. The intervention comprised 12 weeks of three prescribed digital sessions per week: movement and music (*n = *2) and music-only (*n = *1), delivered by an activity coordinator in the care home. Post-intervention interviews with staff and participants were conducted to gain qualitative data on the acceptability of the intervention.

**Results:**

An average of 88% of prescribed sessions were delivered. Pre- to post-intervention intention-to-treat analysis across all participants revealed significant improvements in anxiety, salivary DHEA, fear of falling and loneliness. There were no significant improvements in health-related quality of life, perceived stress, sleep satisfaction or physical function tests, including handgrip strength. Qualitative analysis highlighted benefits of and barriers to the programme.

**Conclusions:**

The digital movement and music intervention was deemed acceptable and delivered with moderate fidelity, justifying progression to a full-scale trial. Although a proper control group would have yielded more confident causal relationships, preliminary psychosocial and biological effects were evident from this trial. To show significant improvements in physical function, it is likely that a bigger sample size providing sufficient power to detect significant changes, greater adherence, longer intervention and/or higher exercise volume may be necessary.

**Trial registration:**

The trial is registered at ClinicalTrials.gov, number NCT05601102 on 01/11/2022.

**Supplementary Information:**

The online version contains supplementary material available at 10.1186/s12877-024-05324-3.

## Introduction

As the demographic shift towards an ageing population accelerates [1, 2], a transition to care home or supported living facilities may become the most viable option for older individuals living alone and requiring high-level supervision and care [3, 4]. In Scotland, UK, care homes provide 24-h support and assistance with daily activities, including personal care, medical monitoring, and social engagement, for older adults who can no longer live independently due to physical or cognitive impairments [5]. These facilities cater to residents with varying levels of need, from those requiring minimal assistance to those needing comprehensive care [6].

The multifaceted challenges confronting older adults residing in long-term care settings, such as limited mobility, limited independent access to outside facilities/activities and lack of social interaction, present barriers to maintaining physical activity (PA) levels [7]. PA has been shown to have significant positive effects on the healthy ageing trajectories of older adults [8], with favourable effects on the endocrine system [9–11], anxiety and depression [12, 13] and physical function [14, 15]. With the rise in technological developments, an increasing number of innovative digital PA interventions have been developed for use in a range of populations and settings [16, 17]. However, research on the effectiveness of digital interventions in promoting PA among older adults in care home settings remains limited [17]. This study aimed to address this issue by investigating the effectiveness of a digital PA intervention specifically designed for the health and wellbeing of older adults resident in care homes with the range of needs and dependencies described above.

Several physiological and psychological challenges are evident in the context of ageing. First, there is a notable change in the functioning of the endocrine system, with the hypothalamic–pituitary–adrenal (HPA) axis being particularly affected, as indicated by changes in the secretion patterns of cortisol and dehydroepiandrosterone and/or its sulphated form (DHEA/S) [18, 19]. This can substantially affect the immune system [20–22]. Second, older adults, whether community-dwelling or in care homes, experiencing reduced social support, chronic disease, and decreased self-esteem are at an increased risk of mental health issues such as anxiety and depression [23, 24], which negatively impacts their wellbeing and quality of life [25, 26]. Finally, a decline in physical function is commonly observed with ageing, increasing older adults’ vulnerability to falling, frailty, rising healthcare costs and premature death [27–29]. While these challenges can have profound negative impacts on older adults, research has demonstrated that PA can counterbalance these challenges and positively influence healthy ageing by improving cortisol and/or DHEA(S) levels [30], anxiety and depression [31], muscle mass, strength, and thus physical function, frailty, and quality of life [32–36] Therefore, PA interventions are a viable approach to mitigating the adverse effects of ageing on the endocrine system, psychological wellbeing, and physical function in older adults.

However, poor adherence to PA programmes is often reported in institutional settings such as care homes [37]. To improve adherence, innovative digital PA interventions have been developed for delivery to older adults, showing promising results to impact overall health, e.g., by improving quality of life [38], muscle mass and physical function [39, 40] and decreasing feelings of anxiety and depression and dementia [41] (for reviews, see [16, 17, 42]). Digital interventions can improve adherence by providing engaging, interactive, and easily accessible content that can be personalised to meet individual needs and preferences, as suggested by the WHO recommendations [43]. However, although most trials showed a positive trend towards the efficacy of digital interventions, better quality evidence is needed considering the heterogeneity in the measures used across studies and to address limitations associated with modest sample sizes and relatively short intervention durations. Further research is warranted to evaluate the effectiveness of digital PA interventions on physiological, psychological and social dimensions of healthy ageing [44, 45]. In particular, the evidence for the effects of digital PA interventions on steroid hormones in older adults is scarce. For example, several studies have noted a lack of quality evidence for the impact of PA interventions in care home residents on anxiety symptoms, cortisol and DHEA(S) [30, 31, 46]. In addition, there is evidence that conducting PA in a group can enhance the effects of the intervention by enhancing social connectedness and reducing loneliness [36, 47, 48]. Thus, interventions combining the digital delivery of PA with a group exercise setting should be examined.

Music, as a common component of group PA delivery, also warrants attention due to the health benefits demonstrated in studies on dance [49–51] and music interventions [52]. The intervention evaluated in this study combined group participation with multicomponent exercises and added a music and singing aspect because music has been shown to positively impact mood, anxiety, and stress, making it an effective tool for improving mental health outcomes [52, 53]. Music can also enhance the enjoyment of PA, increasing adherence and compliance with exercise programmes [54, 55]. Additionally, the use of music may help evoke positive memories and emotions, contributing to overall emotional well-being through reminiscence [56]. However, the effectiveness of music interventions can vary significantly based on individual preferences and the type of PA [57]. The intervention evaluated in this study used a combination of digital delivery and in-person support aimed to address the challenges and maximise the benefits of both group PA and music for older adults in care homes.

To this end, a preliminary study tested the feasibility of a digital music and movement intervention in care homes [58]. Key outcome measures were determined by an advisory group consisting of the research team, care home staff, older adult representatives and representatives from danceSing Care, the company that devised the digital intervention based on important health and wellbeing indicators for these stakeholders and informed by the research literature. The study results determined that such a programme was feasible with recommended adjustments for future implementation [58]. In light of these recommendations, a pilot RCT was designed to investigate whether a 12-week digital music and movement resource, compared to a waitlist control group, would improve some of the same measures from the original study (anxiety, depression, fear of falling, loneliness, quality of life, perceived stress and sleep satisfaction) in addition to measures that are deemed important in previous literature but not possible to measure during the first study (salivary cortisol and DHEA levels and physical function). The primary outcome measures in this study are the physiological markers, specifically salivary cortisol and DHEA levels, and anxiety and depression. These markers were chosen based on previous literature that highlighted their role in reflecting the stress response, overall endocrine health, and older adult well-being, which are indicators of the physical benefits of PA interventions and also where less is known in terms of PA effects than is the case for other e.g., cognitive or depression outcomes in older adults [30, 31].

The primary aim of this pilot RCT was, therefore, 1) to evaluate the efficacy of a digital movement and music intervention within care home settings and its subsequent impact on a range of health and wellbeing outcomes; and 2) to decide whether and how to proceed with a future definitive randomised controlled trial by interpreting specific progression criteria, following the previous feasibility study.

## Methods

### Design

The danceSing Care evaluation trial was designed as a mixed-methods multicentre pilot randomised (1:1), waitlist-controlled trial (RCT) conforming to the CONSORT reporting guidelines for randomised pilot and feasibility trials [59]. Participants were recruited from care home facilities in central Scotland. Data were collected through salivary endocrine measurements, physical assessments, self-report questionnaires, and semi-structured qualitative interviews. Also, process evaluation measures were monitored throughout the intervention period.

Ethics approval was given by the University of Stirling NHS, -Invasive or Clinical Research panel: NICR 2021 3735 3607. This pilot study protocol was registered with the Clinical Trials Register: NCT05601102 on 01/11/2022.

### Procedure, setting and locations

The study occurred at care homes in central Scotland from March to July 2023. Residents capable of giving informed consent were invited to participate in the study after a brief introduction through posters utilised by care home staff and face-to-face interaction with the researchers. Researchers, with the help of care home staff, recruited residents from four Holmes Care Group care homes selected by the Group executive team as representing homes with at least one activity coordinator or similar staff in post, thus available to deliver the intervention. Recruitment started four weeks before the intervention following a live group training and initiation session for the activity coordinators across the Holmes Care Group, including the homes selected to participate in the research. The study design and protocol were explained at this session, including how the wait-list condition would work, along with how adherence data would be collected weekly. Initially, two homes were recruited, but within a week of attempting recruitment, it became apparent that an additional two would be needed to reach the target sample size (see below). Across the four care homes, participant numbers were not evenly distributed due to the varied population size in each care home recruited, the number of residents with the capacity to give consent, and the overall willingness of participants to be engaged in a study. Participants recruited to both intervention and waitlist control groups (1:1 randomisation across all participants recruited) completed baseline and post-intervention questionnaires with the assessors (LDN & EO) in private areas (empty lounges, bedrooms) in their respective care homes to ensure privacy and confidentiality. The intervention lasted for 12 weeks while the waitlist period for the control group was to be about 14 weeks (12 weeks of the intervention plus two weeks of post-intervention data collection prior to receiving the intervention themselves). Physical function testing and saliva sample collection were performed in empty hallways and private rooms. A subsample of seven participants was interviewed two weeks after completing the post-intervention measures to explore additional in-depth intervention effects not captured in the survey, biological, and physical function testing measures. Five care home staff (activity coordinators and/or carers) who facilitated the online intervention were also interviewed in person to share their experiences throughout the process.

### Participants

#### Eligibility criteria

Eligible participants were adults aged 65 or older living in Holmes Care care homes following an agreement with the digital music and movement provider. They needed to be capable of completing a 12-week movement and music programme, providing informed consent, and comprehending the research measures in verbal English, as evaluated by care staff. The care staff were asked to consider the ability of the residents to participate in the study. As the intervention programme is meant to be delivered in care homes inclusive of all residents, no specific physical function cut-off was used as an eligibility criterion other than being able to get out of bed and take part in chair-based exercise as a minimum. The programme was adaptable to each participant's needs, allowing participation either sitting or standing.

Cognitive decline experienced during a previous feasibility study [58] influenced eligibility, particularly regarding understanding research measures among those with cognitive impairment. Due to time constraints and ethical considerations, addressing capacity-related issues for individuals lacking both consent capacity and understanding of measures was not feasible, although they were still able to take part in the music and movement programme.Eligibility assessment followed the use of the British Psychological Society capacity checklist [60], and verbal consent was reaffirmed at each interaction by the researchers. Ineligibility criteria included participation in concurrent clinical trials that could affect this study's outcomes, pre-existing conditions significantly impacting their ability to undergo the intervention, and insufficient English proficiency to engage in measures and intervention due to cognitive or sensory impairments.

#### Sample size

G*power was used to determine the target sample size. The statistical test (ANOVA: Repeated measures, within-between interaction) and the power of the analysis (A priori: compute required sample size) were selected, with α = 0.05 and power = 0.80. The standardised mean difference (SMD) (= 0.80) for the cortisol outcomes was used from a previous systematic review [30] to determine the effect size for this study. This revealed a required total sample size of *n = *16. To account for a possible 20% attrition, the total sample size was inflated to *n = *20. Similarly, the effect size for the anxiety outcome in this pilot RCT was based on an SMD of 0.27 recorded from the previous systematic review [31]. This revealed a required total sample size of *n = *110. To account for a possible 20% attrition, the total sample size was inflated to *n = *132. However, consultation with the care home management suggested that this sample size would not be pragmatic given the high level of cognitive impairment among residents and, thus, the small number of residents eligible for recruitment. Further, the previous feasibility study showed effects in *n = *18 for the psychosocial outcomes, so the target sample in this pilot study was set at 36, based on a general rule of thumb of 30 [61] and accounting for a possible 20% attrition.. Given the feasible sample size, it was determined that individual randomisation (i.e., by participant) would be more pragmatic than cluster randomisation (i.e., by care home), which requires larger sample sizes due to other cluster/site variability [62].

### Intervention

Participants were randomly assigned to participate in a digital movement and music intervention programme from danceSing Care for 12 weeks or a waitlist for 14 weeks before participating. This programme consisted of two movement sessions and one weekly music session, each lasting approximately 20 min. This was the recommended dose suggested in the refinements made from the preliminary feasibility study [58]. These sessions were delivered in a group-based setting, with the digital intervention displayed on a large screen under the supervision of the care home’s activity coordinator in a communal room. The sessions were low-to-moderate intensity, including a warm-up and cool-down period. The intervention gave access to different exercise types, such as strength, flexibility and mobility training, chair-based yoga, breath work, and activities to improve balance and stability for fall prevention. Additionally, certain sessions consisted of singalong activity or themed music. Care home activity coordinator(s) led the sessions and received training on engaging participants and using the digital intervention from danceSing Care programme trainers. The danceSing care programme could be tailored to accommodate the preferences and needs of the participants, with sessions labelled according to their dementia-friendliness and activity coordinators could select the most appropriate session for those taking part. Instructions were integrated into the digital resource to adapt the exercises for sitting or standing postures during movement sessions according to ability, and the music and sing-along sessions were personalised based on the music preferences of older adults. In line with the recommended methods of reporting intervention designs, a TiDieR checklist is detailed in Supplementary File 1.

### Outcome measures

The primary and secondary outcome measures determined by the advisory group and when they were implemented are summarised in Table 1. The primary outcomes were salivary cortisol, DHEA levels, and anxiety symptoms to specifically address gaps identified through review of the literature. Secondary outcomes were fear of falling, loneliness, quality of life, stress, sleep satisfaction, physical function and frailty phenotype. Where possible, brief versions validated in older adults with or without cognitive impairment were used to reduce participant burden. The researchers also piloted the measures on older adults prior to the study start to gauge the level of burden, and during data collection, participants were advised to take a break when they needed to.

**Table 1:** Summary table of outcome measures

Baseline measures	Source	Time point
Demographic information	Self-report	Baseline
**Primary measures**		
Salivary cortisol and DHEA	Assessor	Baseline and post-intervention
Hospital Anxiety and Depression Scale	Self-report	Baseline and post-intervention
**Secondary measures**		
Questionnaires - Falls Efficacy Scale - Dartmouth Cooperative Functional Assessment Charts - Brief UCLA loneliness scale - Perceived Stress Scale - Sleep Satisfaction Tool	Self-report	Baseline and post-intervention
Physical function tests - Short performance battery - Hand grip strength - Fried frailty phenotype criteria	Assessor	Baseline and post-intervention
**Qualitative data**	Semi-structured interviews	Post-intervention
**Progression criteria** - Recruitment rate - Intervention fidelity - Attendance - Retention rate - Safety	Assessor	Post-intervention Monitored during and completed after the intervention

### Questionnaires

#### Demographics

Standardised socio-demographic questions about age, sex, ethnicity, relationship status and education were included to ensure an understanding of the participant pool and contextualise the findings within the backgrounds of the participants.

#### Anxiety and depression

Symptoms of anxiety and depression were measured using the Hospital Anxiety and Depression Scale (HADS) [63]. The HADS consists of two subscales with seven items, each measuring anxiety or depression on a four-point response scale from zero to three, resulting in a maximum score of 21 on anxiety or depression. Specifically, the anxiety subscale covers symptoms of generalised anxiety disorder, whereas the depression subscale focuses on anhedonia [63]. This measure has been validated in older adults with or without cognitive impairment, making it suitable for residents in care homes (Cronbach’s alpha for HADS anxiety = 0.87, HADS depressio*n = *0.81) [64]. The present study’s internal consistencies for anxiety and depression were 0.85 and 0.66, respectively.

#### Falls

The FES-I short validated in older adults (Cronbach’s alpha = 0.92) measured fear of falling while carrying out daily activities such as getting dressed or attending social events [65]. The FES-I is a seven-item scale with responses on a Likert scale from zero (not at all concerned) to three (very much concerned) and total scores ranging from zero to a maximum score of 21. Internal consistency in the present study was 0.93.

#### Health related quality of life

Participants’ health-related quality of life across domains of physical fitness, activities of daily living, feelings, social activities, and change in health and overall health status for the past two weeks was measured with the Dartmouth COOP charts [66] which have test–retest reliability coefficients ranging from 0.67 to 0.82 [67]. This measure has six charts with responses on a five-level ordinal scale ranging from one (no limitation at all) to five (severely limited) for all items except the change in health item, which is reverse scored so that one and five mean much better and much worse, respectively [66]. A high score indicates poorer health-related quality of life, with scores ranging from six to a maximum of 30. Internal consistency in the present study was 0.80.

#### Loneliness

The short-form UCLA loneliness scale (ULS-6) [68] measured feelings of loneliness among participants. The ULS-6 has a Cronbach’s alpha for the reliability of > 0.7 and is composed of six items and response options on a four-point scale (1-never to 4-often), where a high score shows increased subjective feelings of loneliness, and scores range from six to a maximum of 24 [68]. The Cronbach’s alpha in the present sample was 0.85.

#### Perceived stress

The Perceived Stress Scale (PSS) [69] was used to measure how participants experienced their stress during the past month. It consists of ten items with a five-point Likert scale ranging from zero to four that indicates to what extent people perceived stress (depicting low, moderate, and high perceived stress), resulting in a higher total score indicating higher perceived stress from zero to a maximum of 40. Reliability in this study was 0.91.

#### Sleep satisfaction

The National Sleep Foundation’s Sleep Satisfaction Tool (SST) [70] was used to measure sleep satisfaction. It is a nine-item scale scored on Likert response scales (one to four) with a Cronbach’s alpha of 0.87 and a high score suggesting greater sleep satisfaction, where scores can vary from nine to a maximum of 36 [70]. In the present study, the alpha for reliability was 0.90.

### Salivary cortisol and DHEA

A one-point saliva sample for cortisol and DHEA concentration measurement was obtained in the morning by a Salivette cotton wool swab (Salimetrics Ltd., UK) (between 10:00 and 12:30). This timing was specifically selected to mitigate the impact of cortisol's diurnal variation, aiming to capture a more stable period after the morning peak (cortisol awakening response), and time of waking was also recorded. This was optional, requiring consent to opt in. Participants were asked not to eat, drink (other than water), or smoke for two hours before obtaining the salivary samples [71]. The field researcher provided detailed verbal and visual instructions on obtaining the samples to help with compliance. Samples were collected at baseline and post-intervention at the same time of day, and each participant’s waking time and sampling time were recorded in a participant case report form upon sampling completion due to the potential influence of time since waking on cortisol/DHEA concentration.

Saliva samples were collected in Salivette tubes and centrifuged at 4,000 rpm for 5 min, and the supernatant was transferred to Eppendorf tubes (Eppendorf, Hamburg, Germany) for storage at –20 °C until assay. Cortisol and DHEA concentrations were determined using ELISA kits from Salimetrics LLC, USA, following the manufacturer’s protocols. Both assays were conducted in one batch, with samples assayed in duplicate with intra-assay coefficients of < 10%. The assays utilised a competitive immunoassay method, where the unknown cortisol or DHEA in the sample compete with a fixed amount conjugated to horseradish peroxidase for antibody binding sites on a microtitre plate. After incubation and enzymatic reaction, the hormone concentrations were inversely proportional to the optical density read at 450 nm, with a secondary filter correction at 490 to 492 nm. All procedures were executed with precision to prevent contamination.

### Physical function

The short physical performance battery (SPPB) [72] was used to assess physical function among older adults. It comprises three tests: balance, 4-m gait speed, and a chair stand test to assess leg strength. A score between 0 and 4 is assigned for each test, and the three tests are weighted equally. Therefore, the maximum score is 12 points. The cut-off value used to assess poor physical performance is ≤ 8 points, according to the European Working Group on Sarcopenia in Older People (EWGSOP) [73]. The interrater reliability of the SPPB in older adults has been shown to be excellent (ICC = 0.81 to 0.91) [74, 75], and test–retest reliability in people with dementia is also excellent ((ICC = 0.92) [74].

Grip strength was utilised as a surrogate measure of overall muscular strength, which also predicts health and mortality risk [76]. An analogue hand-held dynamometer (JAMAR 5030J1, Sammons Preston) was used to measure the hand grip strength of the dominant hand, taking the best score of three attempts with 20 s rest between the attempts [77]. The test–retest reliability of grip strength in older adults is good (ICC ≥ 0.85) [78].

The Fried frailty phenotype assessment was used to evaluate frailty [79] and is well-established and validated [80]. It considers five criteria: (1) unintentional weight loss measured through the question ‘In the last year, have you lost more than 10 pounds unintentionally (i.e., not due to intentional dieting or exercise)?’ (1 point if present), (2) walk time measured across a 4.57 (15-foot) distance and stratified by sex and height (1 point if above the specified cut-off), grip strength as described above and stratified by sex and BMI (1 point if below the specified cut-off), (3) physical exhaustion measured through two questions from the CES-D [81] asking about feelings or behaviours related to depression in the past week. Participants were asked to reflect on the past week and indicate how often they felt a certain way. They ticked the box in the section corresponding to the frequency of their feelings, with options ranging from “Rarely or none of the time” to “Most or all of the time”. The provided statements, such as “I felt that everything I did was an effort” and “I could not get going,” are indicative of depressive symptoms or feelings (1 point if present), and (4) energy expenditure through the MLTAQ [82] assessment of leisure-time PA and used in conjunction with weight and kcals per activity, (1 point if below the threshold: < 383 Kcals/week for men and < 270 Kcals/week for women). Positive criteria thus score 1 point, dividing participants into three categories. Scores range from 0 (not frail), 1–2 (prefrail), to 3–5 (frail).

### Interviews

The researchers devised the semi-structured interview guide (see Supplementary File 2) in consultation with the study advisory group and focused on the general overview of what participants and activity coordinators thought of the programme, acceptability of the intervention and any benefits they derived or barriers experienced. The interest of participants and activity coordinators in being interviewed was gauged during the baseline data collection and occasional check-in visits to care homes. Based on this, seven residents across three care homes and five activity coordinators from all four care homes agreed. Participants were presented with the participant information sheet and consent forms which included permission to audio record the interview on a password-secured device. Interviews lasted approximately 12 min with 15 to 25 questions, including follow-up questions that probed further into responses. To explore residents’ general thoughts and perceived benefits of the intervention questions such as; “What did you think about the danceSing Care online activities?” and “In what ways have the danceSing care activities impacted your wellbeing?” were used. Additionally, some questions were posed to evaluate potential progression of the intervention. For example, residents were asked “Would you like to continue participating in the danceSing care activities?”. For activity coordinators, the line of questioning was mostly based on the progression criteria/facilitating the intervention and perceived benefits of the intervention to residents and staff. Questions like: “Did residents do complete sessions after starting? If not, why not?” and “Did you manage to engage residents in the 2 Music and Movement + 1 Music sessions a week? If not, why not?” and “What do you think was the impact of danceSing care activities on residents’ wellbeing?” to prompt in-depth responses to support and expand on the quantitative data collected on the benefits and progression of the intervention.

### Delivery outcome—progression criteria

This trial employed a traffic light or Red Amber Green (RAG) approach, with red indicating major problems requiring urgent attention, amber indicating minor problems requiring attention, and green indicating no concern [59]. The criteria were developed in consultation with the advisory group (Table 4). This extended the preliminary realist evaluation that thoroughly documented the feasibility of the delivery and the acceptability of this intervention. The group further assessed the feasibility of specific trial objectives, inclusion and exclusion criteria, and outcomes [58]. Thus, the criteria of this trial were based on recruitment, intervention fidelity, attendance, retention rates, and safety*.* Data were reviewed against the progression criteria, and the research team held regular meetings to discuss progress and concerns.

#### Randomisation

For the allocation of the participants, the Randomization Allocation Software [83] was used for sequence generation in a 1:1 sequence by individual participants. Two researchers enrolled and assessed the participants in the study and allocated ID numbers to each of them. The random allocation sequence was concealed from the researchers at baseline testing as the principal investigator generated the assignment sequence of ID numbers. Details about the intervention and waitlist control allocation groups, including attendance sheets with names of the participants receiving the intervention to enable ease of matching prior to pseudo-anonymisation, were provided to the activity coordinators to return attendance sheets at the end of each week via email.

#### Blinding

Blinding the group allocation is not possible when participants are allocated to an intervention versus waitlist-control group. The researchers taking the measurements were blinded to the allocation of the participants at baseline assessment, but this was not possible at post-intervention assessment. However, the researchers strictly followed approved protocols and guidelines, ensuring the handling of saliva and physical function measurements and questionnaire assessment was as standardised as possible across participants and time points.

### Data analysis

#### Intention to treat analysis of questionnaire, physical function and saliva assay outcomes

Baseline clinical and demographic characteristics were summarised and reported using descriptive statistics. Quantitative data from primary and secondary outcomes were collected, and intention-to-treat (ITT) analyses were performed in IBM SPSS Statistics (version 28.0) to examine the effects of the intervention and the waitlist comparison control group on the primary study outcomes. As stated a priori in the protocol, the intention was to conduct an ITT analysis using repeated-measures between within-groups ANOVA with pre- and post-intervention as the repeated measures and intervention versus waitlist control as between-group variables. However, discussions while collecting follow-up data and through interviewing activity coordinators made it evident that the randomisation protocol had not been adhered to at all sites, resulting in participants in both groups receiving the intervention simultaneously. As a result, a true control group was not established. Consequently, an ITT analysis was conducted on pre-post-intervention change on the whole sample. As a sensitivity analysis, a per-protocol analysis was conducted on the sample with available data, i.e., complete cases only, not ITT.

Estimated effect sizes based on Cohen’s (1988) criteria [84], means, mean differences and their precision (95% confidence interval (CI)) were calculated for continuous data and a count (number, %) for nominal data.

#### Ancillary analysis

To explore if adherence to the intervention had an impact on the wellbeing outcomes, adherence rates for each of the care homes were compared on the change scores across all questionnaires, physical function and physiological metrics by conducting ANOVAs. Adherence rates were grouped as low, moderate, and high for this analysis.

#### Intervention fidelity and attendance

Intervention fidelity was analysed for the duration of the 12-week intervention as the total number of sessions delivered divided by the intended number of sessions (3 per week). Participant attendance was analysed as the number of sessions attended divided by the total number of sessions delivered. The raw fidelity and attendance data were multiplied by 100 to calculate percentage adherence.

#### Qualitative analysis of interviews

Based on the research objectives, a thematic analysis [85] was performed on the qualitative data to identify, analyse, organise, and communicate themes emerging from the interviews. After each interview, one researcher (EO) reviewed the audio recordings and field notes. Audio recordings were transcribed using the Microsoft Word audio recording-to-text feature. Transcripts were cross-checked against the audio recording and all personal details of participants and care homes were anonymised. Anonymised transcripts were then exported into NVivo (version 12) where key words, phrases and quotes relevant to the aims of this study were identified. Themes were then coded from the keywords and phrases found to be consistent throughout the interviews. Themes labelling was mostly in line with components of the progression criteria. The coding process and themes were reviewed independently by another researcher (LDN) to ensure the thematic analysis process was thorough and themes accurately represented the data collected. Thematic analysis was performed independently, but the derived themes were deductive, given that they were linked to the progression criteria; thus, the qualitative results have been presented in association with the progression criteria.

## Results

### Participant flow

**Fig. 1:**
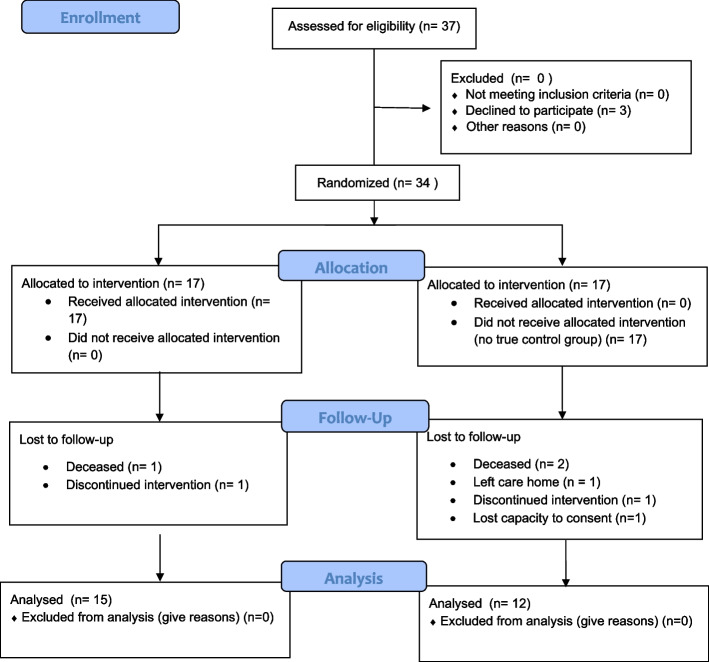
CONSORT Flow Diagram. *Note.* The flow diagram visually represents the number of participants through each stage of the trial, highlighting the reasons for loss to follow-up and the final numbers analyzed

Initially, 37 participants were identified by care home staff as eligible and approached for recruitment from February 2023 to March 2023. The follow-up was completed in July 2023. Thirty-four participants were recruited (seven from each care home except one care home that had 13 participants) and randomised into 17 in the intervention group and 17 in the waitlist control group. Care Home 1 therefore had eight intervention and five control participants; care home 2 had three and four; care home 3 had four and three, and care home 4 had two and five intervention and control participants, respectively. Figure 1 shows the participant flow. In the intervention group, all 17 participants received the intervention. However, one died, and one withdrew, resulting in 15 participants with complete data for the intervention group. Unfortunately, the randomised waitlist control group design was not adhered to. As the interventions took place in the communal living rooms where the waitlist-control group was also present, this group at times, also received the intervention. Further, two participants passed away, one left the care home, one lost the capacity to consent, and one withdrew (*n = *12 with complete data for those originally randomised to be waitlist controls). There were no other missing survey data except for the UCLA Loneliness scale, which one participant chose not to complete. Additionally, four participants did not undergo the physical function tests, and 16 either opted out or could not provide saliva measurements. Therefore, the dataset included 27 participants with pre-post data and 34 for ITT analysis. The subsequent analyses reported here are full sample pre- and post-intervention comparisons. Non-adherence to the delayed delivery of the intervention to the waitlist control group was not evident during the collection of adherence/attendance registers because the attendance of participants on the waitlist was not recorded on the weekly attendance register. This only became apparent in post-intervention data collection and discussion with activity coordinators when arranging testing sessions and interviews.

### Baseline data

Baseline demographics and clinical characteristics for each group are presented in Table 2. Chi-square tests (for categorical data) revealed no significant differences in these characteristics. Most participants were female (59% in the intervention group and 82% in the control group). All participants identified as White: British, Scottish, Welsh, English, Irish, or other. The most common educational attainment was no qualifications (38%).

**Table 2:** Baseline characteristics per group based on randomisation

Variables	Mean (SD) / n (%)		*p*
	**Intervention group** **(*n = *17)**	**Waitlist control group (*n = *17)**	
**Age group**			.31
65–74	3 (18)	3 (18)	
75–84	10 (59)	6 (35)	
85 or over	4 (24)	8 (47)	
**Sex** (Female)	10 (59)	14 (82)	.13
**Ethnic origin** (White)	17 (100)	17 (100)	1.00
**Relationship status**			.25
Single, never married	4 (24)	2 (12)	
Single, divorced or widowed	9 (53)	14 (82)	
Living apart	2 (12)	1 (6)	
Cohabiting	2 (12)	0	
**Highest level of education**			.09
No qualifications	8 (47)	5 (29)	
Completed National 5 s/Standard Grades/GCSE/	5 (29)	4 (24)	
CSE/O-levels or equivalent (at school to age16)			
Highers/Advanced Highers/ AS levels/A-levelsor equivalent (at school to age 18)	4 (24)	1 (6)	
Did not complete National 5 s/Standard	0	4 (24)	
Grades/GCSE/CSE/O-levels or equivalent			
Completed post-16 vocational course	0	1 (6)	
Undergraduate degree or professional qualification	0	2 (12)	

### Primary and secondary outcomes

Table 3 presents the ITT results of all participants’ within-group analyses of pre- and post-intervention changes. For salivary cortisol and DHEA, there was no significant change in salivary cortisol or the cortisol:DHEA ratio, however, salivary DHEA levels showed a significant increase [t(17) = -5.25, *p* < 0.001] although the lower sample size for saliva analyses should be noted. For anxiety symptoms from the HADS, a significant anxiety reduction was observed [t(33) = 2.78, *p =* 0.01].

**Table 3:** ITT analysis of all outcome variables

**Variables**	**n**	**Baseline mean**	**Post-intervention mean**	**Mean difference**	**95% CI**	***p***	**Effect size** (**d**)
Cortisol (ug/dL)	18	0.30	0.40	-0.10	[-.34, .15]	.41	-0.20
DHEA (pg/mL)	18	1455.40	2359.21	-903.81	[-1267.26, -540.36]	< .001*	-1.24
Cortisol:DHEA	18	0.00028	0.00029	0.00001	[-.00011, .00018]	.61	0.12
HADS-Anxiety (0–21)	34	6.00	4.50	1.50	[.40, 2.60]	.01*	0.48
FES (0–21)	34	5.29	3.47	1.82	[.07, 3.58]	.04*	0.36
Dartmouth COOP (6–30)	34	15.97	14.85	1.12	[-.41, 2.64]	.15	0.26
HADS-Depression (0–21)	34	6.44	6.03	0.41	[-.62, 1.44]	.42	0.14
Brief ULS (6–24)	33	12.27	10.64	1.64	[.30, 2.97]	.02*	0.44
PSS (0–40)	34	12.94	11.56	1.38	[-.84, 3.61]	.22	0.22
STT (9–36)	34	29.61	30.57	-0.96	[-2.79, .86]	.29	-0.18
SPPB total score (0–12)	30	4.67	4.67	0.00	[-.73, .73]	1.00	0.00
SPPB balance (0–4)	30	1.87	1.70	0.17	[-.36, .70]	.52	0.12
SPPB gait speed (sec) (0–4)	30	2.17	2.37	-0.20	[-.45, .05]	.11	-0.30
SPPB chair stand (0–4)	30	0.63	0.60	0.03	[-.25, .32]	.81	0.04
Handgrip strength (kg)	30	15.73	16.00	-0.27	[-1.30, 0.76]	.60	0.10
Frailty total score (0–5)	30	2.63	2.63	0.00	[-.20, .20]	1.00	0.00

*FES* Falls Efficacy Scale International (7-item), *Dartmouth COOP* Dartmouth Cooperative Functional Assessment Charts measure of health-related quality of life, *HADS* Hospital Anxiety and Depression Scale, *ULS* UCLA Loneliness Scale, *PSS* Perceived Stress Scale, *STT* National Sleep Foundation Sleep Satisfaction Tool

^*^Significance *p* < 0.05

For the secondary outcomes, significant improvements were observed in loneliness [t(32) = 2.50, *p =* 0.02], and fear of falling [t(33) = 2.11, *p =* 0.04]. However, no significant changes were observed in depression scores, health-related quality of life, perceived stress, sleep satisfaction, SPPB total or individual scores, handgrip strength, or Fried Frailty phenotype.

### Sensitivity analysis

As described above, a sensitivity analysis per protocol on those retained in the study and thus provided data at baseline and post-intervention (*n = *27) was conducted. The summary results in the same format as for the ITT are presented in Supplementary File 3. In brief, the primary and secondary outcomes largely remained consistent between the ITT and sensitivity analyses. However, there were slight differences in the effect sizes for anxiety, fear of falling, and loneliness, with the sensitivity analysis showing slightly larger effect sizes for these outcomes than the ITT analysis.

### Exploring progression to an actual RCT – mixed methods

The outcomes of the progression criteria, which were recruitment, intervention fidelity, attendance, retention rates, and safety, are found in Table 4 with a fuller explanation of the meaning of the criterion in a fuller version in the Supplementary File 5.

**Table 4:** Progression criteria

Progression criteria	Grading	Score	Scoring method	Meaning	Recommendation
**Recruitment rate**	Green	94%	Number of participants recruited (34) divided by target (36)	Slightly lower than expected but feasible	Extending the recruitment period, implementing additional recruitment strategies, increasing advertisement, utilising referrals, and modifying the design to include those without the capacity.
**Intervention fidelity**	Amber	88%	Number of sessions delivered (127) divided by planned sessions (144)	Moderate intervention fidelity	Strategies to enhance fidelity: integrating the intervention into the weekly care home routine, promoting adherence to the randomisation, clear guidelines and training for activity coordinators, and tracking adherence throughout.
**Attendance rate**	Amber	72% overall range: 56–89%	Number of sessions attended divided by total sessions available	Moderate attendance rates with some concerns	Continuous monitoring to identify potential barriers, providing additional support or reminders to improve attendance.
**Retention rate**	Green	94%	Number of participants retained (32) divided by total participants (34)	High retention rates with no immediate concerns	Ongoing efforts: proactive communication, offering incentives or support, and ensuring clear expectations and benefits of participation.
**Safety rate**	Green	None	Number and severity of adverse events reported	No concerns	Continuous monitoring to identify and manage potential adverse events.

### Recruitment rates

The recruitment rate of 34 participants falls slightly below the target sample size of 36. The categorisation as green indicates that while the recruitment rate was slightly lower than anticipated, it is still considered feasible to attain the desired sample size through additional recruitment efforts.

### Intervention fidelity

The activity coordinators in the care homes demonstrated moderate fidelity to the intervention protocol, with some deviations identified in frequency and randomisation. The overall average session delivery rate among the care homes was 88% (127 sessions out of the intended 144 sessions, three sessions each week over 12 weeks) over the intervention period. However, some variability was noted between care homes: one care home delivered 97% of the intended sessions, a second care home provided even more sessions than intended (119%), while a third and a fourth care home provided only 78 and 58% of the sessions, respectively. Overall, this indicated moderate fidelity. Adherence statistics are shown in Supplementary File 4.

The variability of adherence among care homes could have resulted from some barriers encountered during the facilitation of the intervention. From interviews with the activity coordinators (facilitators of the intervention), circumstances in the care home context made delivery of the recommended weekly sessions difficult and, in some cases, impossible. For some care homes, the barriers included a staff shortage, creating additional responsibilities for all staff, poor internet connection and scheduling of the intervention as part of the busy routines in care homes.

For example, activity coordinators said:

*“No, the three sessions a week didn’t hold very well, but that wasn’t because we didn’t want to do it, it was because of certain circumstances. We couldn’t do it. Holidays, people being off sick, staff shortages and things like that. We have to help out wherever we can and, so if we’re not able to do the activities we (activity coordinators) could be put on care and kind of things like that and so that stopped us from doing it, but we always try to manage to do at least once or twice a week. We thought that’s better than nothing”*,

and,

“*The Wi-Fi in here was really shocking and we had someone come out to try and fix it. We bought those Wi-Fi boosters. We bought them for all over here. It’s just [the] building. That was hard. Unless we had someone that had their hotspot from their phone, it constantly cut out. It constantly froze. So that was one of the most difficult things*”,

and,

*“It was time factor for me. Getting everybody together. If they had visitors in, I had to wait till their visitors went and then get them together. And I tried to organise, like a set time, but then when the visitors come in, the visitors are important, so we had to wait longer and longer, but I would say that, and fitting it in around our other activities was the only thing”.*

In addition to contextual barriers, activity coordinators also reported poor engagement from participants and influenced weekly session delivery and frequency. For some participants, poor engagement was due to cognitive impairment. For others, it was poor physical health, individual preferences, low motivation, and enthusiasm towards the intervention. To explain the challenges, activity coordinators said:

*“I think for some of them, it was as a result of their dementia or things like that. They have a shorter attention span and sometimes when they’re sitting there, they will all of a sudden not realise why they’re there. We have (Participant A) in particular who would always say, “what am I doing here? What am I doing here?” but after we’d explained to her about a good few times what we were doing, she calms down. So some of them were just like that”,*

and,

*“It depended on the mood of the day. You could get one resident that is absolutely amazing one time and then on another day they just don’t want to do anything”,*

and,

*“I think some of them (participants) did enjoy the programme and some didn’t. I think we struggled with the movement sessions. There were a few of them that didn’t really want to do any kind of dances or a lot of the movements, but they loved the singing ones, they absolutely loved them”*

One participant also said,

*“Well, I’m just not as able as I was. I think it’s a struggle to me now, even getting up and about. It just changed overnight. I like to do it but sometimes I’m just not able, like last night, I just wasn’t well at all.”*

Despite the deviations in frequency per week, it is noteworthy that once a session was conducted, the activity coordinators adhered to the planned intervention components, including duration and intensity. This suggests a level of commitment to maintaining fidelity within the delivered sessions. Additionally, from the interviews, activity coordinators reported that the intervention has gradually been integrated into the care home routine and expressed interest in its continuity. The statement below from one activity coordinator showed positive acceptability and satisfaction with the intervention:

*“We are going to continue using it, we are going to continue it as much as we can. I mean if we don’t get it all the time, if we’re only able to maybe do the once or twice a week or even if it only goes down to one, it’s still going to get done. It’s part of the curriculum now, so it’s going to stay”.*

However, it is essential to re-emphasise that none of the care homes followed the waitlist control group randomisation process as instructed in the protocol, indicating low fidelity in that aspect.

To improve adherence and participation among participants, activity coordinators adopted mechanisms that made facilitation easier in their respective care homes, irrespective of the difficulties at the onset and the challenges during the intervention period. For some care homes, the activity coordinators joined in the sessions to help motivate and maintain enthusiasm. For other care homes, it was adding it to the schedule and telling visitors ahead of time when sessions would start. In line with this, activity coordinators said:

*“Sometimes, not always, it was easy to get them back to continue the sessions. We just kind of started dancing with them and kind of danced them back towards where they were. It was kind of sneaky but we liked it”,*

and,

*“I think when we (activity coordinators) are up moving about more instead of sitting and doing the exercises they’re more inclined to get involved. Even though I’m sweating at the end of it, they’re more inclined to enjoy it. I think they get a good laugh out of it”,*

and,

*“Well, I spoke to some of their families, and they said don’t worry about it just come and get her (participant) if she’s to go to that (intervention session). It’ll be fine and so the families were really good”,*

and*,*

*“Probably like doing it with them. I wasn’t like just letting them do it by themselves I was actually involved and liked engaging with them”.*

### Attendance of participants

Although the intended controls received the intervention, the activity coordinators provided no data on these participants’ retention or attendance rates. Therefore, the data provided below, and in Supplementary File 4, consist of the residents initially randomised in the intervention group. The mean participant attendance rate out of the possible total number of sessions delivered, thus available to them in one care home, was 82%. In another care home, it was 60%; in the third care home, it was 89%; and in the fourth care home, it was 56%. Attendance of participants was influenced by several factors, including personal, social and the intervention design as described above. In addition, the interviews from both residents and activity coordinators revealed that participants’ engagement in the programme was motivated by their personal history and the memories the intervention brought back, the conversations it started and the opportunity to do something different. Participants said:

*“Well, it’s easy dancing, so you can do that. I quite liked getting a bit of fun out of it”,*

and,

*“I liked the singing, I liked us all singing together and people up dancing”,*

and,

*“It was past the morning and it was something to do, something to listen to rather than just sitting up here doing crossword. It brought us all together and we all got up, we danced and it was a good atmosphere. I thought it brought everybody together and so it was a good change from just sitting in here doing nothing”,*

and,

“*I used to be a singer. I am good at singing and so I was happy to join the programme*”

In support of how participants accepted the intervention, activity coordinators said:

*“I must admit they liked Alan the singer. When we put that up, they get really involved in. The exercises they’ve done but not with the same gusto as the singing”,*

and,

*“Oh definitely, because sometimes they’ll come and say, “what time are we doing that thing at the day”? They don’t say danceSing. It is good that they looked forward to it”,*

and,

*“…because even when they’re dancing with you, they start chatting away to you as well, and when they’re doing certain songs, they’ll say, “I did this such a time”. They remember when they’ve heard a song, which is kind of cute. The songs brought back good memories”.*

Participants in these care homes demonstrated commitment to completing the intervention or following the protocol as instructed. With attendance rates at 72% overall, the moderate adherence rates suggest that participants actively engaged in the intervention activities as intended, increasing the likelihood of achieving the desired outcomes.

### Retention rates

During the 12-week intervention period, one participant in the intended intervention group discontinued the intervention. This signifies that most participants were retained throughout the study. The retention rate of 94% signifies a high retention rate showing most participants were engaged and committed to the study.

Other than engagement, commitment and other factors that influenced participant attendance, the high retention rate in this study can also be attributed to the perceived impact of the intervention on their physical and psychosocial wellbeing. Participants and activity coordinators described the benefits of the intervention as boosters that fuelled participation and continuity throughout the intervention period. Specifically, one participant said:

*“I think I am getting a little bit stronger. The fear is going away, and I want to try things”*

Additionally, activity coordinators explained the impact of the intervention by saying:

*“It was good. It was good because they (participants) improved a lot. Like, I had one resident downstairs, if she’s not doing something, she’s overthinking. When she wasn’t in the programme, she was overthinking but because she distracted herself with the dance and singing, it made her happy because she was quite depressed. So, it was good for some residents obviously. It distracted them from overthinking by doing something else and by moving about. So, I think it’s a really good programme”,*

and,

*“It helped them to move a lot more and obviously coming down and socialising in a group”,*

and,

*“It was good. It was getting a lot of them together including those that weren’t involved in the research. It was good to see everyone being engaged and taking part in the programme. It brought them together”,*

and,

*“Well, they were a lot happier, they were upbeat, they had a laugh… And they spoke about it afterwards. They were very enthusiastic, put it that way”,*

### Safety rates

Throughout the intervention period, no significant adverse events were reported among the participants. This indicates a favourable safety profile, with no adverse events of significant concern observed during the study.

### Ancillary analyses

ANOVAs were conducted between the care homes based on adherence rates, at the request of the care home organisation, to explore whether adherence (as a proxy for dose) influenced outcomes. Groups were classified as: low (58%), moderate (78%), and high adherence (97% and 119%). There were no significant differences between adherence groups) on change scores for any of the study outcomes, except for the Dartmouth COOP health-related quality of life scores [F(2,33) = 4.52, *p =* 0.02]. *Post-hoc* tests revealed no change in quality of life pre-to-post-intervention in the low adherence group whereas both the moderate (*p =* 0.01) and high (*p =* 0.04) adherence groups significantly differed from the low group and showed slight improvement in this score.

## Discussion

The primary aim of this pilot mixed method study was twofold: 1) to evaluate the efficacy of a digital movement and music intervention within care home settings and its subsequent impact on a range of health and wellbeing outcomes; and 2) to decide whether and how to proceed with a future definitive randomised controlled trial. The main findings revealed increased salivary DHEA levels and reduced anxiety, loneliness, and fear of falling. The study’s qualitative insights also provided a deeper understanding of the intervention’s progression criteria. Participants expressed enjoyment and appreciation for the opportunity the intervention provided to come together, fostering a sense of community and shared experiences.. However, the implementation of the intervention faced challenges, including time constraints due to care home routines, understaffing, and technical issues such as unreliable Wi-Fi connections. The trial’s analysis was complicated by care homes’ non-adherence to the waitlist control condition, resulting in all participants receiving the intervention simultaneously.

The increase in salivary DHEA levels observed aligns with studies highlighting the positive effects of PA on older adults’ endocrine function, specifically the improvement of DHEA levels even in small samples [30, 86, 87]. Such increases have been associated with improved immune function, enhanced cognition, and reduced risk of chronic diseases in older adults [88–90]. The lack of significant change with PA in cortisol levels contrasts with previous literature showing that PA improved cortisol output [46, 91–93]. However, this could be explained by this study’s lack of statistical power and/or the fact that training intensity should exceed 60% VO_2_ max to impact on the HPA axis [94, 95] and the current digital PA intervention was of low to moderate intensity. It is also suggested that the exercise effects on the HPA-axis differ from person to person and depend on the person’s personal choice of PA, needs, values and circumstances [30, 96, 97]. Objective metrics such as heart rate monitors could provide more accurate insights into the programme’s intensity, enabling the activity coordinator to tailor the programme in real-time to the participant’s needs.

Further, a single daily measurement of saliva was opted for, a decision driven by practical and financial constraints. While a single sample in the morning is often and reliably used to assess diurnal secretory activity to assess within-subject variations over a certain period in an older population [98], this approach does not comprehensively reflect the intricate diurnal fluctuations of cortisol nor account for inter-individual day-to-day and intra-individual variations, especially within a smaller sample [99, 100]. Future studies could integrate multiple measurements across consecutive days to gain a more nuanced understanding of cortisol dynamics and its relationship with DHEA [101].

There was a reduction in anxiety symptoms post-intervention, reinforced by qualitative findings of participants experiencing diminished ‘fear’. This aligns with literature emphasising the benefits of PA on mental health parameters, specifically anxiety [31, 102]. PA has been shown to reduce anxiety symptoms through multiple mechanisms: improved brain circulation, increased release of endorphins, and enhanced self-efficacy [103]. The care home environment, where residents might often feel confined or limited in their autonomy [104], could heighten feelings of anxiety. Thus, interventions that encourage movement and social interaction might offer considerable benefits in reducing such feelings.

The non-significant findings for depression symptoms contrast with some previous studies. While numerous studies have highlighted the antidepressant effects of PA [105, 106], the results did not indicate a significant change in depression levels post-intervention. It is worth considering the intervention’s duration, intensity, or the specificities of the current cohort, which may account for the lack of observed change [31]. Previous research suggests that psychological benefits may be observable even with low doses of PA such as once per week over a relatively short period [31]. Our previous study showed some positive effects on anxiety, depression, loneliness, perceived stress and sleep satisfaction over 12 weeks with 3 × music and movement and 1 × music only sessions per week as the recommended dose [58], and the present study showed effects for loneliness, anxiety and fear of falling with just 2 × music and movement plus 1 × music only sessions per week. It is therefore possible that longer or more intense PA would be necessary to influence depressive symptoms, or that effects would be more likely to emerge for those with higher levels of symptoms at baseline. Although changes in anxiety were statistically significant, in this cohort, the baseline mean scores for both anxiety and depression fell within the "normal" range. This could imply that participants had relatively low levels of these symptoms to begin with, making significant reductions more challenging to achieve. This supports the hypothesis of a potential floor effect contributing to the absence of a significant change in depression scores [64]. Additionally, the aetiology and chronicity of depressive symptoms in a care home population might be multifactorial [107], requiring interventions beyond PA and music.

One interesting finding was the disparity between psychological wellbeing improvements and the lack of substantial effect sizes in physical function tests and frailty markers. The improvements in wellbeing markers echo the idea that psychosocial factors, such as social engagement in group-based PA, play a critical role in older adults’ mental health [108, 109]. On the other hand, the lack of improvements in physical function and frailty was surprising, given substantial evidence of the benefits of PA on physical function and frailty in other research [14, 110–113]. This intervention might not have improved these measures due to the limited duration, frequency and intensity of the programme. Additionally, the reduced sample size for these specific measures likely impacted the ability to detect significant changes, highlighting the need for larger sample sizes in future studies. However, the programme did provide a range of low-to-moderate intensity types of PA including strength, flexibility and mobility training, chair-based yoga, breath work, and activities aimed at improving balance and stability for fall prevention. This variety meant the consistency of the intervention was variable across care homes, given the choice available. However, previous research has shown that different types of physical activity yielded varied effects on frail institutionalised older adults' cognitive state, functionality, and general health [111] thus supporting the activity approaches in the present intervention. Others have reported a correlation between regularly completing activities ranging from low intensity walking to more vigorous PA and resistance exercise and reduced risks of functional limitations, disability, and cognitive impairments in older people [14, 113]. Given this, future implementations of the present intervention as well as newly developed interventions need to employ a range of strategies to ensure overall PA increases, including both aerobic and strength and balance aspects of PA. Alternatively, the lack of effects on physical function may be due to a range or factors such as the lack of a control group, small sample size, or sensitivity of the measures. Further, the time needed to observe changes in frailty markers may be longer than that for psychological markers, potentially requiring more extended intervention periods to detect significant results. Although positive effects on frailty and physical function have been observed in PA studies as short as six weeks in similarly small samples, these studies typically involved more intensive resistance training with three strength-building sessions per week and increasing intensity [36]. This suggests that it is not just the duration of an intervention that influences whether physical frailty effects are observed, but also the type and intensity of PA [114].

Feedback from the participants highlighted the added value of the music and dance components. These elements have been successfully integrated into previous intervention programmes for older adults, improving balance, physical function, muscle strength and endurance [49–51, 115, 116]. Concurrently, the profound emotional resonance of music, with its capacity to evoke cherished memories and stimulate dopamine release, has been previously identified as a powerful tool for enhancing mental wellbeing [52, 117–119]. This was also noted during the interviews, where participants highlighted their enjoyment of the music components, suggesting these played an essential role in the positive outcomes observed. Beyond their direct benefits, the music and dance elements may also have acted as motivators, potentially increasing participation and commitment to the programme, as studies in older adults have found that adding music to PA can increase enjoyment and, as a result, participation in PA programmes [54, 55]. This, along with the social interaction of group activities, can enhance mental, emotional, and physical health [120]. Reminiscence, through familiar songs and dance, can evoke positive memories and emotions, further contributing to emotional wellbeing [121]. These elements could potentially lead to better adherence and more significant outcomes [58]. This approach justifies the need for a future full-scale RCT to explore the broader applicability and benefits of such interventions in care settings. However, this would need to incorporate testing of the separate effects of reminiscence-based music on top of group-delivered PA, to fully elucidate the extent to which this component contributes additively or synergistically to the overall positive effects on health and wellbeing observed in the present study.

The qualitative analysis revealed recurrent barriers and facilitators influencing intervention efficacy, mirroring findings from other studies, such as logistical challenges and staff enthusiasm [122, 123]. Participant feedback emphasised the acceptability of the programme. However, some suggested tailoring for enhanced engagement, such as using motivational facilitators to make the programme fun and sociable with relevant short-term benefits, aligning with previous findings [124] or strategically incorporating the programme part of the weekly care home schedule, with advance notice of session commencement times. Organisational culture in care homes and activity coordinators’ support levels critically impacted intervention fidelity, underscoring the need to foster a conducive organisational environment [125, 126].

### Strengths and limitations

This study has several strengths, such as adopting a mixed-methods approach, lending depth and granularity to the insights, and facilitating a holistic understanding of the intervention’s impact and implementation. Further, the use of progression criteria as guidelines helped identify potential challenges and develop solutions to improve the quality and feasibility of the intervention [127]. However, it is important to acknowledge certain limitations. The control group’s non-adherence to the waitlist condition caused a deviation from the protocol and compromised the ability to make direct comparisons, forcing reliance on within-group pre-post-intervention analyses. This provides a strong rationale for using cluster-randomisation in a future RCT and adjusting the sample size accordingly. Further, while not uncommon in these settings, recruitment challenges and resulting small sample size may have introduced a selection bias and produced underpowered results. Therefore, effect sizes are presented alongside p-values and it is recommended to interpret these results cautiously. Nevertheless, the consistency of the observed outcomes in ITT and sensitivity analyses makes the results more reliable. However, the variance in effect sizes between the two analytical approaches for specific outcomes underscores the significance of accounting for participant adherence and attrition in future research. This variance emphasises that participant engagement and adherence factors may moderate the intervention’s potential benefits. Further, the study’s scope was limited to 12 weeks. A more extended observation period might provide deeper insights into the sustainability and long-term effects of the intervention. This concern was also raised in a preliminary systematic review regarding the effects of PA on cortisol and DHEA(S) in older adults. However, adopting a longer duration was out of scope for the present study due to the duration of initial commitment Holmes Care agreed to and limited timing of the PhD projects this was part of.

### Future directions to progress to an RCT and future research

This study emphasises the potential benefits of PA interventions in care homes, especially when enriched with music and dance components. The study’s application of the Red Amber Green (RAG) progression criteria offers valuable insights for future trials. The recommendations about recruitment, programme fidelity, attendance and retention rate and safety are outlined in Table 4. However, several critical future directions should be emphasised. First, the experience with non-adherence from the control group underscores the significance of robust protocol adherence mechanisms. Future studies should prioritise establishing strict guidelines and transparent communication channels between researchers and involved care home staff. Regular audits, complemented by frequent check-ins, can ensure the consistent application of the study protocol across care homes, which would be possible with a fully powered and staffed clinical trials unit-supported RCT. Alternatively, as suggested above, cluster-randomisation could be used.

Second, the care home staff play an integral role in the successful deployment and fidelity of interventions. Hence, ongoing training, mentorship, and support are of paramount importance. Future studies should focus on comprehensive initial training sessions followed by regular refresher courses and workshops, although programme instructors did have several strategies in place to encourage engagement and provide support, such as an initial training session, ‘connect and reflect’ sessions and an in-person visit with social media footage, motivational e-mails, and a private Facebook group. Implementing fidelity checklists and conducting video reviews of sessions can also help in monitoring and maintaining the intervention's consistency and integrity. Third, the ancillary analyses showed that adherence levels in care homes significantly affected the Dartmouth COOP health-related quality of life scores, demonstrating improvement only in moderate and high adherence groups, contrasting with a stable score in the low adherence group. These findings accentuate the importance of future studies adhering to the prescribed three sessions per week or more to maximise intervention benefits. Further, gathering information on participants’ comorbidities might have provided further insight into variation in the intervention’s effectiveness and why certain outcomes positively changed, and should be measured in future studies. Finally, the reassuring safety profile, high participant attendance and retention rates observed pave the way for subsequent larger-scale RCTs sufficiently powered for exploring the intervention’s efficacy. Although the study design utilised a generalised approach to the digital movement and music intervention, the responses among participants highlighted the potential benefits of personalisation. Future research should consider methods for individualised customisation, ensuring that interventions are tailored to each participant’s unique needs, preferences, and physiological responses. Employing adaptable digital platforms, offering a range of activities within the digital PA sessions or interactive features that encourage personalised engagement could facilitate this customisation.

## Conclusion

The study contributes insights into implementing digital PA (music and movement) interventions in care homes. Preliminary significant findings over time emphasise the programme’s positive influence on resident wellbeing, as evidenced by enhanced salivary DHEA levels, reduced anxiety and other wellbeing markers. Specific recommendations regarding recruitment and programme fidelity strategies were made to proceed to a subsequent full-scale RCT. For recruitment rate, additional efforts such as extending the recruitment period, intensifying recruitment efforts, and modifying the design to include those without the capacity to consent can be considered to achieve the required number of participants. Regarding intervention fidelity, strategies to enhance adherence to the planned frequency and randomisation process were proposed, including making the intervention part of the weekly care home routine, providing clear guidelines, and training for activity coordinators. Addressing identified barriers through additional support and allocating a dedicated ‘exercise room’ were also recommended. Further, more proactive adherence tracking could help in early identification and address any deviations from randomisation protocols. These results underline the potential for this digital PA intervention to shape subsequent research and the practical application of similar interventions in care settings, fostering a more multi-dimensional and evidence-driven approach to care home interventions.

## Supplementary Information

Supplementary Material 1.

## Data Availability

The datasets used and/or analyzed during the current study are available from the corresponding author on reasonable request.
