# Supplementary material for: Digital music and movement intervention to improve health and wellbeing in older adults in care homes: a pilot mixed methods study
Source: BMC Geriatr. 2024 Sep 4;24:733. doi: 10.1186/s12877-024-05324-3 (PMC11373285; doi:10.1186/s12877-024-05324-3)
Supplement: Supplementary file 1 — Supplementary Material 1. [file 12877_2024_5324_MOESM1_ESM.docx]

**Supplementary Files**

Supplementary file 1: TiDieR checklist

Supplementary file 2: Semi-structured interview guide

Supplementary file 3: Table 3: Per protocol analysis of all outcome variables for participants with complete data (n = 27)

Supplementary file 4: Table 4: Intervention fidelity and attendance of participants

Supplementary File 5: Table 5: Progression criteria

**Supplementary file 1**: TiDieR checklist

| TiDieR | Checklist requirement | Protocol description |
| --- | --- | --- |
| 1. | Brief description of the intervention | danceSing are programme: digital music and movement resources to improve multidimensional health markers in older adults in care homes |
| 2. | Rationale and theory of the elements essential to the intervention | - Physical activity interventions, including multi-component (chair-based) exercises or dancing, and music therapies have been shown to improve multidimensional health markers in older adults - Increasingly, innovative digital resources have been developed to influence physical activity in care homes |
| 3. | Materials | For the participant and activity coordinator:   - Information folder about the danceSing care evaluation   For the activity coordinator:   - Training day and protocol documentation, including how the danceSing care programme works - Personal username and password to enter the digital danceSing care resources |
| 4 | Procedures | - Randomisation to intervention or waitlist group (1:1) - Participants were contacted before the pre-intervention testing to inform them of group allocation - Intervention participants received the intervention from week 1 to week 12 |
| 5. | Who provided | - All sessions were delivered by the activity coordinator of the care home - All activity coordinators received an initial training session on how to engage participants in the programme during the trial and how to work with the digital resources |
| 6 | Mode of delivery | All sessions were group-based. Digital resources were provided on a big screen, under the supervision of the activity coordinator of the care home |
| 7. | Location of delivery | - Activity coordinator training sessions took place at the care homes - danceSing care session took place in a communal room in the care home |
| 8. | Intervention duration, intensity and dose | - One music and up to 2 movement sessions each week for 12 weeks - The sessions were of low-to-moderate intensity, at least 20 minutes, with a short warming-up and cooling-down part |
| 9. | Tailoring | - The sessions are labelled by a dementia friendly-tag, ‘all stages’ or ‘mild-to-moderate’ - Movement sessions are labelled with tags like ‘chair fitness’ and ‘standing fitness’ and instructions are provided to adapt a standing or sitting posture during the sessions. - The music and sing-along sessions are tailored to the music preferences of older adults |
| 10. | Modifications | Adaptations to the programme were made after the feasibility study [(Ofosu et al. 2023)](https://sciwheel.com/work/citation?ids=14767203&pre=&suf=&sa=0&dbf=0) – reduction in the number of sessions as noted above |
| 11. | How well planned | - Attendance and adherence registers were given to the activity coordinators to be returned to the researchers every week - Researchers contacted the activity coordinator if the files were not sent through in time or if the attendance or adherence was not as expected |
| 12 | Delivered as planned | The intervention was delivered as planned in the intervention group |

**Supplementary file 2:** Semi-structured interview guide

**Supplementary file 3: Per protocol analysis of all outcome variables for participants with complete data (n = 27)**

| Variables | n | Baseline mean | Post-intervention mean | Mean difference | 95% CI | p | Effect size (d) |
| --- | --- | --- | --- | --- | --- | --- | --- |
| Cortisol (ug/dL) | 18 | 0.30 | 0.40 | -0.10 | [-.34, .15] | .41 | -0.20 |
| DHEA (pg/mL) | 18 | 1455.40 | 2359.21 | -903.81 | [1267.26, -540.36] | <.001* | -1.24 |
| Cortisol: DHEA | 18 | 0.00028 | 0.00029 | 0.00001 | [-.00011, .00018] | .61 | 0.12 |
| HADS-Anxiety (0-21) | 27 | 6.30 | 4.41 | 1.89 | [.53, 3.25] | .01* | 0.55 |
| FES (0-21) | 27 | 5.56 | 3.26 | 2.30 | [.09, 4.50] | .04* | 0.41 |
| Dartmouth COOP (6-30) | 27 | 15.96 | 14.56 | 1.41 | [-.52, 3.34] | .15 | 0.29 |
| HADS-Depression (0-21) | 27 | 6.30 | 5.78 | 0.52 | [-.80, 1.83] | .43 | 0.16 |
| Brief UCLA loneliness (6-24) | 26 | 12.50 | 10.42 | 2.08 | [.41, 3.75] | .02* | 0.50 |
| PSS (0-40) | 27 | 12.70 | 10.96 | 1.74 | [-1.08, 4.56] | .22 | 0.24 |
| STT (9-36) | 27 | 30.26 | 31.47 | -1.21 | [-3.53, 1.11] | .29 | -0.21 |
| SPPB total score (0-12) | 24 | 4.83 | 4.83 | 0.00 | [-.92, .92] | 1.00 | 0.00 |
| SPPB balance (0-4) | 24 | 1.96 | 1.75 | 0.21 | [-.46, .88] | .53 | 0.13 |
| SPPB gait speed (sec) (0-4) | 24 | 2.17 | 2.42 | 0.25 | [.56, .06] | .11 | -0.34 |
| SPPB chair stand (0-4) | 24 | 0.71 | 0.67 | 0.04 | [-.32, .40] | .81 | 0.05 |
| Handgrip strength (kg) | 24 | 0.96 | 0.92 | 0.04 | [-.11, .19] | .58 | 0.12 |
| Frailty total score (0-5) | 24 | 2.67 | 2.67 | 0.00 | [-.25, .25] | 1.00 | 0.00 |

*Note:* FES: Falls Efficacy Scale International (7-item), Dartmouth COOP: Dartmouth Cooperative Functional Assessment Charts measure of health-related quality of life, HADS: Hospital Anxiety and Depression Scale, UCLA: University of California, Los Angeles, PSS: Perceived Stress Scale, STT: National Sleep Foundation Sleep Satisfaction Tool. * significance *p* < 0.05

**Supplementary file 4:** **Intervention fidelity and attendance of participants**

| Number Sessions Offered Per Week | | | | | | | | | | | | | | | |  |
| --- | --- | --- | --- | --- | --- | --- | --- | --- | --- | --- | --- | --- | --- | --- | --- | --- |
| Care Home | **1** | **2** | **3** | **4** | **5** | **6** | **7** | **8** | **9** | **10** | **11** | **12** | **Total** | **% Sessions Offered** | **Average sessions per week** | **% Attendance of participants** |
| 1 | 3 | 3 | 3 | 4 | 2 | 3 | 3 | 2 | 3 | 2 | 0 | 0 | 28 | 78% | 2 | 82% |
| 2 | 2 | 0 | 2 | 2 | 2 | 4 | 0 | 2 | 1 | 2 | 2 | 2 | 21 | 58% | 2 | 60% |
| 3 | 4 | 3 | 3 | 3 | 3 | 3 | 3 | 4 | 3 | 4 | 6 | 4 | 43 | 119% | 4 | 89% |
| 4 | 2 | 3 | 3 | 3 | 3 | 3 | 3 | 3 | 3 | 3 | 3 | 3 | 35 | 97% | 3 | 56% |

Note: Care home 1 = Heatherfield, 2 = Larkfield View, 3 = Beechwood, and 4 = Almond View care home.

**Supplementary File 5: Progression criteria**

| **Progression criteria** | **Cut-off scores for each progression criterion** | **Grading with traffic light system** | **Meaning** | **Recommendations** |
| --- | --- | --- | --- | --- |
| **Recruitment rates** | - Green (No Concern): Recruitment meets or exceeds the expected target. - Amber (Minor Problem):  The cut-off for amber is set at achieving 70-90% of the expected recruitment target. - Red (Major Problem): Recruitment falls significantly below feasibility, with less than 70% of the expected target achieved. | Green: Slightly lower than expected but feasible (94%) | If the recruitment rate is lower than expected but still feasible to achieve the required number of participants, additional efforts can be made to improve recruitment. | Additional efforts such as extending the recruitment period, implementing additional recruitment strategies (such as reaching out to more care homes or collaborating with other organisations), intensifying recruitment efforts (e.g., increasing advertisement or utilising referrals), modifying the design to include those without the capacity to consent can be considered. |
| **Intervention Fidelity** | - Green (No Concern): High fidelity to the intervention protocol, with minimal or no deviations observed. - Amber (Minor Problem): Moderate fidelity with some deviations from the protocol. The amber threshold is defined as adherence to 70-90% of the key elements of the intervention. - Red (Major Problem): Poor fidelity with significant deviations. Less than 70% adherence to the key elements of the intervention. | Amber: Moderate intervention fidelity (88%) | This indicates room for improvement and adjustments in future implementations to enhance fidelity and ensure closer adherence to the intervention protocol. Attention should be given to addressing the identified deviations and barriers to ensure the integrity of the intervention delivery. | First, strategies can be implemented to enhance fidelity to the planned frequency, such as making it part of the weekly care home routine. Second, efforts should be made to promote adherence to the randomisation process by providing the activity coordinators with clear written guidelines and training. Addressing the identified barriers, such as providing additional support and training or allocating a separate ‘exercise room’, may also improve fidelity. Third, tracking adherence rather than calculating it at the end would help identify if randomisation protocols need addressing early on. |
| **Attendance rate** | - Green (No Concern): High attendance rates, with participants attending 75% or more of the scheduled sessions. - Amber (Minor Problem): Moderate attendance, with participants attending 60-75% of the sessions. - Red (Major Problem): Low attendance, with less than 60% attendance. | Amber: Moderate attendance rates (56-89% depending on the care home, with 72% overall attendance) | This category signifies participants completed the intervention or followed the protocol with minimal deviations or non-compliance, and there are some concerns regarding adherence. | Continuous attendance monitoring remains essential to identify any potential barriers or challenges that may impact attendance in the future. Ongoing assessment can help inform adjustments to the study protocol or provide additional support or reminders, if necessary, to improve attendance further. |
| **Retention rate** | - Green (No Concern): High retention rates, with 80% or more of the participants remaining in the study until its conclusion. - Amber (Minor Problem): Moderate retention, with 60-79% of participants retained until the end of the study. - Red (Major Problem): Low retention, with less than 60% of participants retained. | Green: High retention rates | This indicates that 80% or more participants were successfully retained with no immediate concerns and suggests that the study effectively maintained participant involvement and minimised attrition during the 12-week intervention period. | Ongoing efforts such as proactive communication with participants, offering incentives or support, and ensuring clear expectations and benefits of participation can further enhance retention rates in future studies. |
| **Safety rates** | - Green (No Concern): No significant adverse events reported. - Amber (Minor Problem): Minor adverse events reported, but these do not significantly impact the overall safety of the intervention. The amber threshold is defined as less than 5% of participants experiencing minor adverse events. - Red (Major Problem): Significant adverse events reported, affecting more than 5% of participants, or any severe adverse event, regardless of frequency. | Green: No significant adverse events were reported, with no concerns. | This indicates that the study can proceed as planned without substantial safety issues. | Continuous monitoring allows for identifying and appropriately managing potential adverse events, even if they are minor or expected. |
